# Detection and measurement of alpha-amylase in canine saliva and changes after an experimentally induced sympathetic activation

**DOI:** 10.1186/s12917-017-1191-4

**Published:** 2017-08-22

**Authors:** María Dolores Contreras-Aguilar, Fernando Tecles, Silvia Martínez-Subiela, Damián Escribano, Luis Jesús Bernal, José Joaquín Cerón

**Affiliations:** 1 Interdisciplinary Laboratory of Clinical Analysis (Interlab-UMU), Veterinary School, Campus of Excellence Mare Nostrum, University of Murcia, 30100, Espinardo, Murcia, Spain; 2 Department of Food and Animal Science, School of Veterinary Medicine, Universitat Autònoma de Barcelona, 08193, Bellaterra, Barcelona, Spain

**Keywords:** Salivary alpha-amylase, Dog, Saliva, Sympathetic activation

## Abstract

**Background:**

Salivary alpha-amylase (sAA) is considered a biomarker of sympathetic activation in humans, but there is controversy regarding the existence of sAA in dogs. The hypothesis of this study was that sAA exists in dogs and it could change in situations of sympathetic stimulation. Therefore, the aims of this study were: 1) to demonstrate the presence of alpha-amylase in saliva of dogs by Western-Blot, 2) to validate an spectrophotometric method for the measurement of sAA activity and 3) to evaluate the possible changes in sAA activity after the induction of an ejaculation in dogs which is known to produce a sympathetic activation.

**Results:**

Western-Blot demonstrated a band in dog saliva specimens between 60 kDa and 50 kDa, similar to purified sAA. The spectrophotometric assay validated showed an adequate inter- and intra-assay precision, and a high correlation coefficient (*r* = 0.999) in the linearity under dilution study. sAA median activity significantly increased just after ejaculation compared with just before the ejaculation (2.06-fold, *P* = 0.005).

**Conclusions:**

This study demonstrated the existence of alpha-amylase in saliva of dogs and that this enzyme can be measured by a spectrophotometric assay. In addition, results showed that sAA increase after a sympathetic activation and could be potentially used as non-invasive biomarker of sympathetic activity in this species.

## Background

Salivary alpha-amylase (sAA) is considered in humans as a biomarker of sympathetic activation [1, 2], increasing not only in exercise [3], but also after psychological stressors [4]. This biomarker is measured in saliva, which can be obtained easily and in a non-invasive way [5, 6].

sAA has been also evaluated in other animal species such as pigs [7], Bonobos and other simians [8], horses [9] and sheep [10]. However, in dogs despite the growing interest in the evaluation of stress [11–13], there is controversy regarding the existence of sAA. Some authors described a lack of sAA [14, 15], whereas others identified sAA by sodium dodecyl sulphate polyacrylamide gel electrophoresis (SDS-PAGE) following liquid chromatography coupled with tandem mass spectrometry (LC-MS/MS) approaches [16]. In addition, to the authors’ knowledge there are no validated assays for alpha-amylase measurement in dogs saliva, as well as it is unknown whether sAA increases in those situations producing sympathetic activation in this species.

The hypothesis of this study was that sAA exists in dogs and it could show changes in situations of sympathetic stimulation. To test this hypothesis, firstly a Western-Blot was performed to detect the presence of alpha-amylase in canine saliva. Then, a method for sAA measurement based on its enzymatic activity was validated in dogs, and the changes of sAA activity after an experimental model of sympathetic activation consisting on the induction of an ejaculation were studied.

## Methods

### Saliva sampling

Saliva specimen was collected introducing a small sponge around the mouth until it was thoroughly moist. Sponges were placed in collection devices (Salivette, Sarstedt, Aktiengesellschaft & Co, Nümbrecht, Germany) and stored with ice until arrival at the processing laboratory, where devices were centrifuged at *3.000 g* for 10 min at 4 °C. Saliva was transferred into 1.5 mL Eppendorf tubes and stored at −80 °C until analysis that were performed 1 week later.

### SDS-PAGE and Western-Blot

Twenty five μg of total protein from a pool integrated by four canine saliva specimens obtained from the sympathetic activation pilot situation with high sAA activity and 10 μg of total protein from a concentrated saliva specimen from a healthy dog were used for this approach. To concentrate saliva, an Amicon 50 K molecular weight cut-off (MWCO) filter (Millipore Corp., Billerica, MA) was used as previously reported [17] because the molecular mass of sAA is higher than 50 kDa [18]. Purified human sAA (ab 77,875, Abcam, Cambrigde, UK) and a human saliva specimen (10 μg protein each) were used as positive controls. Total protein was determined using the bicinchoninic acid method [19]. Proteins were separated in mini polyacrylamide gels containing 0.1% (*w*/*v*) sodium dodecyl sulphate (SDS-PAGE), with a separating gel prepared in 10% (*w*/*v*) and a stacker gel prepared in 4% (*w*/*v*) according to the methodology described by Laemmli [20], stained with 1% *w*/*v* coomassie Brillant Blue for 5 min, followed by distaining with 5% *v*/v ethanol 7% *v*/v acetic acid for 24 h. Western-Blot were carried out as described previously [21] using an indirect detection. Proteins separated in SDS-PAGE were transferred to nitrocellulose membrane (Bio-Rad Laboratories Inc., Hercules, CA, USA). Rabbit polyclonal antibody against human sAA (ab 173,163, Abcam, Cambridge, UK) at 1:500 dilution were used as primary antibody, while horseradish peroxidase (HRP)-conjugated goat polyclonal antibody anti-rabbit (ab 6721, Abcam, Cambrigde, UK) at 1:2000 were employed as secondary antibody, which one was detected using Pierce ECL2 kit (Pierce, Thermo Fisher Scientific, USA) and Thyphoon 9410 scanner (GE Healthcare, Wilmington, MA, USA).

### sAA enzymatic assay

sAA activity was determined by a commercial kit (alpha-Amylase, Beckman Coulter Inc., Fullerton, CA, USA) using the International Federation of Clinical Chemistry and Laboratory Medicine method [22]. This assay is a spectrophotometric assay that uses 4,6-ethylidene (G7)-p-nitrophenol (G1)-alpha-D- maltoheptaoside (G7PNP) as a substrate of the enzyme. The intermediate product of substrate hydrolysis reacts with a-glucosidase, giving p-nitrophenol as the final product of the reaction. The rate of p-nitrophenol formation was directly proportional to the alpha-amylase activity of the sample and could be determined by measuring the absorbance at 405 nm. The assay was adapted to an automatic analyzer (Olympus UA600, Olympus Diagnostica GmbH, Ennis, Ireland), according to the manufacturer’s indications regarding reagent and specimen volumes.

### Analytical validation of sAA enzymatic assay

The following characteristics were evaluated for the analytical validation of the method:

- Reproducibility. Three canine saliva specimens from the sympathetic activation pilot situation described below with low, medium and high sAA activity were used. Intra-assay precision was evaluated by measuring 6 times each of the selected specimens in the same analytical series. Inter-assay precision was calculated by measuring each of the specimens once a day for 5 days; the possible effect of repetitive thawing and freezing was removed by storing of the samples in separate vials (aliquots) and using a new one for each measurement. Results were expressed as the coefficient of variation (CV): standard deviation divided by mean of the replicates times 100%.
- Accuracy. It was evaluated by linearity under dilution (indirect method), because no reference assay is available for sAA activity in dogs. Two specimens with different sAA activity were serially diluted (75%, 50%, 25%, 12.5%) with deionized water. The results were compared with those expected by linear regression analysis.
- Limit of detection. It was defined as the lowest concentration of the analyte that could be distinguished from a specimen of zero value, and it was calculated on the basis of data from 13 replicate determinations of the zero standard (deionized water) as mean value plus three standard deviations.
- The lower limit of quantification. It was calculated as the lowest sAA activity that could be measured above the limit of detection with a CV <20%. A saliva specimen was serially diluted in deionized water and each dilution was analyzed in five replicates in the same run. CVs for each dilution were estimated as previously described.

### Experimental model of sympathetic activation

The model for sympathetic activation consisted on semen collection in 10 healthy male beagles of a research colony of the Animal Resources Center of Murcia University (average age = 13.0 ± 3.9 months). All of them were inside kennels in-groups of two individuals. Semen specimens were taken by digital manipulation [23], using a latex semen collection cone (artificial vagina) and 15 mL sterile centrifuge tubes. Artificial vagina was washed with water between each dog to avoid sperm contamination. Saliva specimens were collected at least 30 min before the ejaculation at basal time (TB), just starting the ejaculation (T-0), just after ejaculation (T + 0), and 30 min later (T + 30). The heart rate was also measured at TB, T + 0 and T + 30, and the dogs’ sexual behaviour during the semen collection (pelvic thrusting, engorgement of the bulbus glandis and full erection) was evaluated [23]. The experimental situation lasted from 8:30 to 10:35 a.m. To perform the entire procedure, each dog was taken out of its original kennel and passed to a close empty kennel.

To guarantee the presence of the second fraction (sperm-rich), semen specimens were evaluated as Toro-Montoya [24] describes.

### Statistical analysis

Arithmetic means, medians, CVs and linear regression analyses were calculated using routine descriptive statistical procedures by spreadsheet (Excel 2000, Microsoft Corporation, Redmond, Washington, USA). Normality was evaluated by using Shapiro-Wilk test showing non-parametric distribution in sAA activity results, therefore they were transformed logarithmically by applying the formula ln x = ln (x + 1) before the statistical analyses [25, 26]. Analyses of variance of repeated measures with Fisher’s LSD tests were used to determine if the values at different times were significantly different. The significance level used in each case was *P* < 0.05. These statistical analyses were calculated using Graph Pad Prism 6 (GraphPad Software, San Diego, CA, USA).

## Results

### SDS-PAGE and Western-Blot

**Fig. 1:**
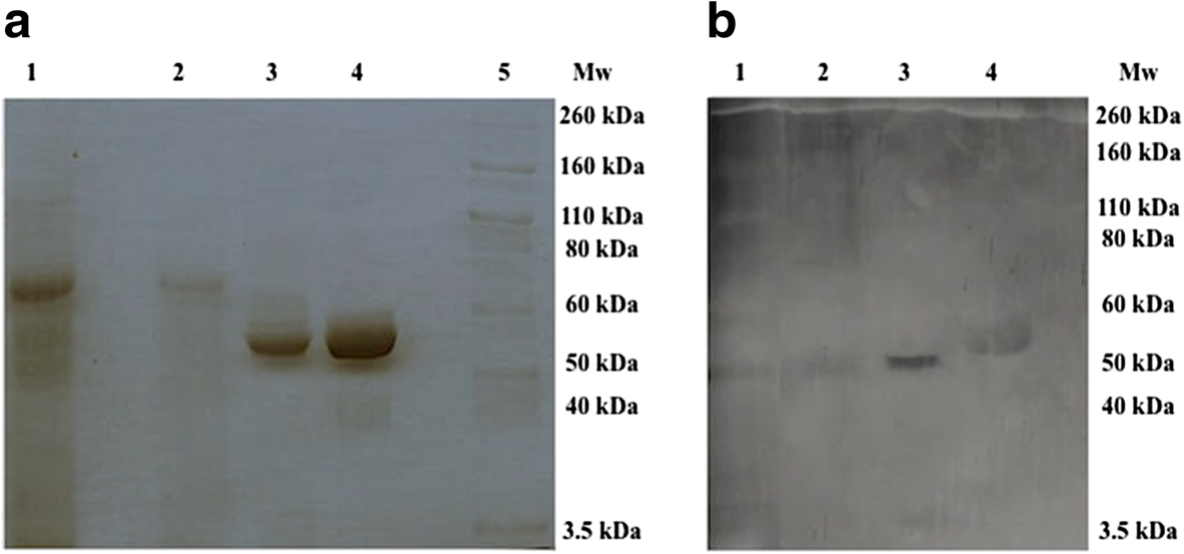
SDS-PAGE (**a**) and Western-Blot (**b**). Lane 1: 10 μg dog saliva specimen concentrated; Lane 2: 25 μg pool of canine saliva; Lane 3: 10 μg human saliva; Lane 4: 10 μg purified human sAA (ab 77,875, Abcam, Cambrigde, UK); Lane 5: Molecular weight markers (Novex Sharp Pre-Stained, Invitrogen, Carlsbad, California)

SDS-PAGE and Western-Blot results are shown in Fig. 1. In SDS-PAGE (Fig. 1a), human saliva and purified human sAA (ab 77,875, Abcam) showed two bands between 60 kDa and 50 kDa, while an undefined band could be intuited in concentrated dog saliva. This band did not appear in the pool of canine non-concentrated saliva. Western-Blot (Fig. 1b) showed a band with high intensity between 60 kDa and 50 kDa in the human saliva specimen and the purified human sAA (ab 77,875, Abcam). This band also appeared in both canine specimens, although the intensity was lower compared to human saliva.

### Analytical validation of sAA enzymatic assay

**Fig. 2:**
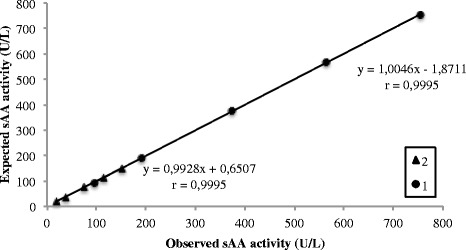
Linearity under dilution of two specimens with different activity of salivary alpha-amylase (sAA): 755.2 and 152.1 U/L, specimen 1 and 2 respectively. The ‘x’ expressed sAA activity measured and ´y´ sAA expected level at the particular dilution. r = Pearson r-value of linear correlation

**Fig. 3:**
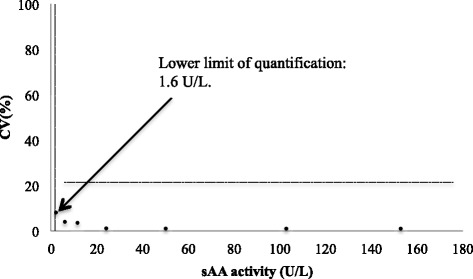
Limit of quantification graph for detection of salivary alpha-amylase (sAA) activity. Horizontal line show the highest coefficient of variation (CV) accepted (20%) for the limit of quantification calculation. The vertical line reflects the analytical limit of detection (1.6 U/L)

Results from the precision study are shown in Table 1. Intra-assay and inter-assay CVs (%) for sAA were <4% for specimens with low, medium and high sAA activity. The linearity under dilution study yielded a coefficient of correlation of 0.999 for both, specimen 1 and 2 (Fig. 2). In both specimens, slopes [0.99 (0.98; 1.02) and 1.00 (0.98; 1.01), respectively] were not significantly different from one, and intercepts [−2.11 (−11.63; 7.40) and 0.03 (−1.28; 1.35), respectively] were not significantly different from zero. The limit of detection, as well as lower limit of quantification, was set at 1.6 U/L, since CVs of all dilutions tested were below 20% (Fig. 3).

**Table 1:** Intra- and inter-assay coefficients of variation (CVs) in saliva specimens with different alpha-amylase activity

	Intra-assay		Inter-assay	
Specimens	Mean (U/L)	CV (%)	Mean (U/L)	CV (%)
Low	40.3	2.00	33.4	2.60
Medium	160.0	2.27	130.0	1.38
High	765.7	1.27	656.3	3.47

### Experimental model of sympathetic activation

**Fig. 4:**
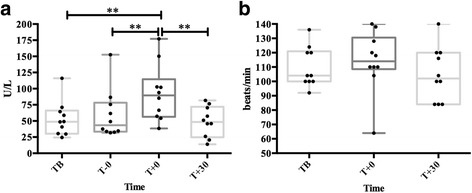
Salivary alpha-amylase (sAA) activity (**a**) and heart rate (**b**) during an experimental model of sympathetic activation in dogs. TB: basal time; T-0: just starting the ejaculation; T + 0: just after ejaculation; T + 30: 30 min later. The plots show medians (line within box), 25th and 75th percentiles (boxes), min and max values (whiskers) and individual values (points). Asterisk indicates statistically significant difference (**P < 0.01) between times

All dogs showed erection signs (pelvic thrusting and engorgement of the bulbus glandis) and ejaculated. The ejaculation’s average duration was 50.7 ± 22.2 s. sAA median activity (Fig. 4a) showed significant increases at T + 0 when compared with TB (1.84-fold, *P* = 0.004) and T-0 (2.06-fold, *P* = 0.005). Heart rate (Fig. 4b) did not show any significant change.

## Discussion

Western-Blot findings corroborated the results obtained with the enzymatic assay and indicated that alpha-amylase exists in dog saliva, reacting with the sAA antibody used in this study and producing a band between 50 and 60 kDa as occurs in humans. This band was observed in both concentrated and un-concentrated canine saliva with similar intensity between them, but having lower intensity compared to human saliva probably due to a lower immunological cross-reactivity of the antibody against canine alpha-amylase or to the lower alpha-amylase concentration that exists in canine saliva compared to human. The later is in accordance with the SDS-PAGE findings and with enzymatic activity results obtained in our study, since sAA enzymatic activity in human saliva could reach ranges of 200–300 × 10^3^ U/L after psychological stress [5, 25] or even 1441 ± 262 × 10^3^ U/L after post intense exercise [3], which is much higher that the median values (89.5 U/L) observed in the dogs of our study. The low alpha-amylase concentration in saliva was the possible reason why Chauncey et al. [14] could not quantitate its levels in the dog. It is interesting to point out that the values of alpha-amylase in saliva of dogs were lower than the values that can be found in serum of healthy dogs (our reference interval for serum alpha-amylase is 250–1300 U/L).

Analytical validation of the sAA enzymatic method showed an acceptable intra- and inter-assay imprecision, being in all cases lower than 15%, which is the limit recommended for method validation [27]. In addition, it is lower than the within-subject biologic variation reported by Ricos et al. for sAA in human [28]. The assay showed high correlation coefficients (*r* = 0.999) and a good linearity (slopes were not significant different of one, and the intercepts were not of zero) in serially diluted saliva pools. These results demonstrate that the activity–response relationship is similar in the calibration curve and the specimens, and the sample matrix and calibrator diluent did not affect the response in signal [29]. All the measurements made by sAA enzymatic assay in this study were higher than the limits of detection and quantification of the assay (set at 1.6 U/L), with a minimum recorded value of 15.2 U/L.

In this study, the induction of an ejaculation was selected as a model of sympathetic activation to evaluate sAA behavior, since it has been described that ejaculation is caused by stimulation of the sympathetic nerves which stimulate bulbocavernosus and ischiocavernosus muscles to expel semen and prostatic fluid by peristaltic contractions [23]. A prompt sAA raise at the end of the ejaculation in dogs was observed. These results could indicate that sAA increases after sympathetic activation in this species.

Heart rate has been also proposed as a marker of sympathetic nervous system activation [30, 31]. However, there were not significant increases in heart rate in our study. This is in agreement to Beerda et al. [32], who did not find a significant response in dogs to different stress stimulus (sound blasts, short electric shocks, a falling bag, an opening umbrella and two forms of restraint) when heart rate was evaluated.

It is described that alpha-amylase in human saliva is produced in the salivary glands and not diffuse into saliva from blood [25]. Probably in dogs could occur the same process. However the very low although measurable alpha-amylase activity found in dog’s salivary glands and other organs, but the very high activity found in pancreas [33, 34] could open the possibility that alpha-amylase in dog saliva could be produced in the salivary gland (salivary isoenzyme) but also be partially of plasmatic origin (plasmatic isoenzyme) by diffusion from plasma into saliva as it is reported for other salivary enzymes [35].

The limited number of dogs employed in this study, and the fact that they were only male beagles from a research colony, makes difficult to extrapolate these results to the entire canine population or to real conditions. Therefore, this report should be considered as a pilot one and further studies in dogs of different sexes, breeds and living conditions, as well as using others stimulating models should be performed in order to fully evaluate the possible use of saliva alpha amylase as a stress marker.

## Conclusions

This study demonstrates that there is alpha-amylase in saliva of dogs and validates a reliable spectrophotometric assay to measure this enzyme in this specie with a good precision, sensitivity and accuracy. In addition, it reports a sAA activity increase after an experimental model of sympathetic activation in the dog and suggests that sAA could be potentially used as non-invasive biomarkers of sympathetic activation in this species.

## Abbreviations

CV: Coefficient of variation
G7PNP: 4, 6-ethylidene (G7)-p-nitrophenol (G1)-alpha-D- maltoheptaoside
HRP: Horseradish peroxidase
LC-MS/MS: Liquid chromatography coupled with tandem mass spectrometry
MWCO: Molecular weight cut-off
sAA: Salivary alpha-amylase
SDS-PAGE: One-dimensional sodium dodecyl sulphate polyacrylamide gel electrophoresis
